# 

**Published:** 1904-12

**Authors:** 


December, 1904. VOL. XXV. No. 12.

### THE INTERNATIONAL DENTAL JOURNAL.

JAMES TRUMAN, D.D.S., EDITOR,

PHILADELPHIA.

### Summary of Contents.

(*For details, see p. 2.*)

ORIGINAL COMMUNICATIONS.

REPORTS OF SOCIETY MEETINGS.

REVIEWS OF DENTAL LITERATURE.

EDITORIAL.

BIBLIOGRAPHY.

CURRENT NEWS.

*$2.50 a Year, in Advance. Single Copies, 25 Cents.*

PUBLISHED MONTHLY BY

The International Dental Publication Company,

227–231 SOUTH SIXTH ST., PHILADELPHIA.

EDITORIAL OFFICE, 4505 CHESTER AVENUE, PHILADELPHIA, PA.

PRINTED BY J. B. LIPPINCOTT COMPANY, PHILADELPHIA.

Entered at the Post-Office at Philadelphia, and admitted for transmission through the mails at second-class rates.

